# Chicago Medical Register and Directory for 1872–3

**Published:** 1872-11

**Authors:** 


*NOW READY—The Chicago Medical Register and Directory for* 1872–3. Published under the revision of the following Committee: Dr. J. W. Freer, President Rush Medical College; Dr. N. S. Davis, President Chicago Medical College; Dr. W. H. Byford, President Woman's Hospital Medical College; Dr. G. C. Paoli, President Chicago Medical Society; Dr. D. B. Trimble, President Chicago Association of Physicians and Surgeons; Dr. D.W. Young, President Illinois State Medical Society. Edited by Dr. T. Davis Fitch, Permanent Secretary Illinois State Medical Society, 296 W. Monroe St.; and Dr. Norman Bridge, 267 W. Monroe St.

It describes the character, object, and field of operations of the Hospitals, Infirmaries, Dispensaries, Charitable Institutions, and

Asylums of the entire State of Illinois. Also, the Medical and other Scientific Societies and Associations, with full Lists of Membership. It contains, also, announcements of the Schools of Medicine and Pharmacy. Full directions are given respecting the means of reaching and obtaining the benefits of these various institutions. Full information is given to medical students regarding the colleges, their location, the curriculum of studies, the fees, and requirements for graduation.

There is given a *List of Physicians* in good standing in Chicago and vicinity, and in the larger towns of the State, with residence, office, and office hours; also a *List of Dentists* regularly graduated as D.D.S. or M.D., or who are members of a Dental Society; also a *List of Veterinary Surgeons* who are regularly educated; also a *List of Regularly Educated Pharmacists.*

The *Register and Directory* will be mailed free of postage on receipt of $2.00.

Address "MEDICAL REGISTER," 296 W. Monroe St., Chicago, Ill.; or W. B. KEEN, COOKE & Co., 113 and 115 State Street, Chicago.

***Prof. Freer,***

Having rebuilt his residence on the North Side, will hereafter be found there. Correspondents and friends will govern themselves accordingly. His No. is 224 Ontario Street, between Clark and Dearborn.

